# A Class IIb Bacteriocin Plantaricin NC8 Modulates Gut Microbiota of Different Enterotypes *in vitro*

**DOI:** 10.3389/fnut.2022.877948

**Published:** 2022-06-30

**Authors:** Jiaqian Pu, Shuting Hang, Manman Liu, Ziqi Chen, Jiayi Xiong, Yongquan Li, Hongchen Wu, Xiaodan Zhao, Shuxun Liu, Qing Gu, Ping Li

**Affiliations:** Key Laboratory for Food Microbial Technology of Zhejiang Province, College of Food Science and Biotechnology, Zhejiang Gongshang University, Hangzhou, China

**Keywords:** bacteriocin, plantaricin NC8, *in vitro* fermentation, gut microbiota, short-chain fatty acids, enterotypes

## Abstract

The gut microbiota is engaged in multiple interactions affecting host health. Bacteriocins showed the ability of impeding the growth of intestinal pathogenic bacteria and modulating gut microbiota in animals. Few studies have also discovered their regulation on human intestinal flora using an *in vitro* simulated system. However, little is known about their effect on gut microbiota of different enterotypes of human. This work evaluated the modification of the gut microbiota of two enterotypes (ET B and ET P) by the class IIb bacteriocin plantaricin NC8 (PLNC8) by using an *in vitro* fermentation model of the intestine. Gas chromatography results revealed that PLNC8 had no influence on the gut microbiota’s production of short-chain fatty acids in the subjects’ samples. PLNC8 lowered the Shannon index of ET B’ gut microbiota and the Simpson index of ET P’ gut microbiota, according to 16S rDNA sequencing. In ET B, PLNC8 enhanced the abundance of *Bacteroides*, *Bifidobacterium*, *Megamonas*, *Escherichia-Shigella*, *Parabacteroides*, and *Lactobacillus* while decreasing the abundance of *Streptococcus*. *Prevotella*_9, *Bifidobacterium*, *Escherichia-Shigella*, *Mitsuokella*, and *Collinsella* were found more abundant in ET P. The current study adds to our understanding of the impact of PLNC8 on the human gut microbiota and lays the groundwork for future research into PLNC8’s effects on human intestinal disease.

## Introduction

There are trillions of microbial cells in the human body, and their coordinated functions are considered crucial for human survival. Most of these microbes are found in the intestines, where they form the complex microbial community that known as the gut microbiota (1), which, in humans, begins to grow in infancy and matures within the first years of life (2–4). Members of the gut microbiota include archea, bacteria, fungi, and viruses, all of which have developed complicated nutritional connections and one other and with their human host (5). The abundant and diverse members of the human gut microbiota play an important role in human health by assisting in the breakdown of food substances to liberate nutrients that would otherwise be inaccessible to the host, promoting host cell differentiation, protecting the host from colonization by pathogens, and stimulating/modulating the immune system (6). Bacteriocins are relatively small antimicrobial peptides synthesized by the ribosomes of most known bacteria (e.g., Firmicutes, Bacteroidetes, Actinobacteria, and Proteobacteria; 7) that can alter the gut microbiota populations. Nisin supplementation to the daily feed of broiler chickens reduced potentially pathogenic flora, such as *Clostridium perfringens* and *Enterobacteriaceae*, in the jejunum and cecum, positively impacting the gut microbiota and significantly reducing bacterial fermentation in the jejunum, according to Kierończyk et al. (8). During the first 4 h after intraperitoneal injection into mice, nisin F inhibited the growth of specific species, such as *Staphylococcus*, *Staphylococcus aureus*, and *Listeria* spp., and had a stabilizing effect on the bacterial flora in the gastrointestinal tract (9). Furthermore, adding nisin to rabbits’ drinking water for 28 days lowered the prevalence of *Staphylococci*, coliforms, *Pseudomonas*, *Clostridium*, and *Enterococcus* in the rabbit intestine (10). In an independent study, Ke et al. discovered that adding antimicrobial peptides (nisin and cecropin) and herbs (*Penthorum chinense*) to carps’ daily diets significantly altered their gut microbial community, with nisin and *P. chinense* both decreasing the relative abundance of Proteobacteria and increasing the relative abundance of Bacteroidetes (11). When broilers and rabbits were fed the bacteriocin plantaricin K, the abundances at the genera level of *Lachnoclostridium*, *Streptococcus*, and *Ruminococcaceae*- UCG-013 were dramatically upregulated while the abundance of *Bacteroides* was downregulated (12). These literature studies demonstrate that bacteriocins particularly nisin can change the gut microbiota.

Although the influence of bacteriocins on animal intestinal microorganisms is well understood, bacteriocins have not yet to been used in the area of human medicine; hence the specific effects of bacteriocins on the human gut microbiota remain unknown. The previous studies had mostly used *in vitro* fermentation systems where feces and bacteriocins were co-fermented to investigate the effects of bacteriocins on gut microbiota *in vitro*. As early as 2009, Pediocin A [a bacteriocin produced by lactic acid bacteria (LAB) *Pediococcus pentosaceus* FBB61] was found to modulate intestinal microflora metabolism in swine *in vitro* intestinal fermentations (13). Subsequently, Bactofencin A was discovered to have a relatively subtle influence on intestinal communities, with a potentially positive impact on anaerobic populations such as *Bacteroides*, *Clostridium*, and *Bifidibacterium* spp. in an *in vitro* fecal fermentation system in adults (14). And Umu et al. also used fecal slurry cultures to study *in vitro* effects of bacteriocins on the gut bacterial populations of infants and found that *Bifidobacterium* had reduced and *Lactobacillus* had increased in nisin-treated fecal slurry, while GarML reduced *Bifidobacterium* at only high concentrations (50 μg/ml; 15). Recent studies also show that using an *ex vivo* model of the colon, purified nisin had the ability to selectively deplete *C. difficile* in a fecal microbial environment and established the minimum concentration at which this occurs while having a minimal impact on the composition of the microbiota (16).

All indicators point to bacteriocins having a potentially positive influence on human gut microbiota, yet different people have distinct enterotypes, which means their gut microbiota is various as well. People who eat a diet high in animal proteins and fats and have a fatty gut type have a *Bacteroides* enterotype, whereas those who eat a high-carbohydrate diet for a long time have *Prevotella* enterotype. Sometimes, the enterotypes *Ruminococcus* and *Bacteroides* are overlapped and found to be indistinguishable (17). According to discriminating genus, Arumugam et al. (18) grouped 33 samples from diverse populations into three distinct robust clusters: *Bacteroides* (enterotype 1), *Prevotella* (enterotype 2), and *Ruminococcus* (enterotype 3; 19).

The PLNC8, which consists of two peptides, PLNC8α (29 amino acids), and PLNC8β (34 amino acids), belongs to the class IIb bacteriocins. PLNC8 proved efficient at eliminating *S. aureus* and counteracting its inflammatory and cytotoxic effects, whether taken alone or in combination with low amounts of gentamicin (20). Furthermore, a 1:1 molar ratio of PLNC8α and PLNC8β demonstrated effective antibacterial action against *S. aureus* and *Staphylococcus epidermidis* (21). In our previous study, PLNC8α or PLNC8β alone or at a 1:1 molar ratio as PLNC8 showed minimum inhibitory concentrations (MICs) against *Micrococcus luteus* CGMCC 1.193 of 3.2, 1.6, and 0.8 μM, respectively, which suggests that PLNC8α and PLNC8β alone possessed independent activity but were the most active in combination (22). The combination of PLNC8α and PLNC8β heterologously expressed in *Escherichia coli* showed the highest inhibitory activity against *Salmonella spp.*, and both had half-maximal inhibitory concentrations (*IC*_50_) of approximately 0.15 μg/ml (23). On the basis of previously validated inhibition concentrations, this work chose 3 μM as the low concentration and 30 μM as the high concentration.

The effect of bacteriocins on intestinal bacteria of various enterotypes has not been described in earlier studies. The majority of the research available focused on the effects of polysaccharides on intestinal flora of various enterotypes, with roughly 11 in the NCBI database. This study combined PLNC8 and adult feces for *in vitro* (24) fermentation in order to explore the influence of bacteriocins on the gut microbiota of various enterotypes. This work categorized the 8 subjects into two enterotypes based on the definition of enterotypes: enterotype 1 (ET B) and enterotype 2 (ET P). Bacteriocins were linked to enterotypes for the first time in laboratory data. The impact of PLNC8’s synthesis of short-chain fatty acids (SCFAs) on gut flora has also been investigated.

## Materials and Methods

### Materials

Hangzhou evergreen Co., Ltd provided the acetic acid. Trypsin, Yeast extract, L-cysteine, Hemoglobin solution, Sodium chloride, Calcium chloride solution, KH_2_PO_4_, K_2_HPO_4_, and Magnesium sulfate solution were purchased from the Shanghai Sangon Biotech Bioengineering Co., Ltd. The reagents used were all analytically pure. PLNC8 was mixed in equimolar proportions from the high purity oligopeptide PLNC8α and PLNC8β. And PLNC8α and PLNC8β were synthesized by the GL Biochem (Shanghai) Ltd.

### Collection of Samples

Morning fresh feces were collected from eight healthy subjects from Key Laboratory for Food Microbial Technology of Zhejiang Province. These subjects had not taken antibiotics in the past 3 months. They ate traditional Chinese food, had healthy dietary habits, and did not smoke or abuse alcohol. This research was open to all subjects and our research was approved by the Ethics Committee of Zhejiang Academy of Agricultural Sciences (Ethics Committee Agreement Number: 20212203-04).

### *In vitro* Fermentation of PLNC8

Fresh adults’ feces were fermented with synthetic PLNC8 *in vitro* (25). The 0.8 *g* of fresh fecal sample were mixed with pretreated PBS (autoclavation, 0.1 mM, pH 6.8) to prepare a 10% (W/V) fecal homogenate suspension. After filtration, the fecal supernatant was transferred to a centrifuge tube and placed in an anaerobic work station (Do Whitley Scientific) for the later use. The fecal supernatant (500 μl) was inoculated in the autoclaved YCFA medium (26; 5 ml) containing the testing samples (NC83, NC830) and fermented at 37°C for 24 h. The fermentation broth (2 ml) was centrifuged in a sterile centrifuge tube at 12,000 rpm for 5 min. The supernatant was absorbed and stored in a separate EP tube in –20°C refrigerator for the detection of SCFAs, while the precipitate was used for DNA extraction and subsequent analysis of bacterial structure. This work set a low concentration of PLNC8 (NC83, 3 μM) and a high concentration of PLNC8 (NC830, 30 μM) as the experimental group and a blank group (F) as the blank control.

### Short-Chain Fatty Acids Analysis

Before the SCFAs were determined, feces samples were collected and processed. The processing was as described in section “*in vitro* Fermentation of PLNC8.” Gas chromatography (GC) was used to identify SCFAs in samples from the mimic intestinal system. Propionic acid, butyric acid, acetic acid, valeric acid, and isobutyric acid among the SCFAs were detected in the mimic intestinal system and crotonic acid was used as internal standard. After homogenization of 50 mg of colon tissue with 1 ml of 6% phosphoric acid solution, all homogenates were transferred to an injection vial and sealed with a further 1 ml of the internal standard solution. The GC conditions were as follows: temperature program started at 80°C, ramped up to 220°C and held for 0.5 min; high-purity helium was the carrier gas, with a flow rate of 1 ml/min and a split ratio of 10:1; the injection temperature was 200°C, and the flame ionization detector temperature was 250°C, followed by a static headspace injection with an incubation temperature of 85°C and an incubation time of 30 min; the injection needle temperature reached 95°C, and 1 ml of sample was injected. In the selected ion monitoring mode of mass spectrometry, the ion source temperature was 250°C. The areas of resolved peaks on extracted ion chromatogram were recorded to plot the standard curve. Then, the peak area ratios were used to compute the concentration of each component in each sample using the standard curve.

### Effect of PLNC8 on Gut Microbiota Structure

Bacterial genomic DNA was obtained from fermented fecal samples using the QIAamp^®^ PowerFecal^®^ DNA kit (cat 51604) following the manufacturer’s instructions (QIAGEN, Hilden, Germany). The integrity of DNA was confirmed by 1% agarose gel electrophoresis (27). Bacterial DNA was extracted and preserved on dry ice, and immediately sent to Biomarker Technologies for high-throughput sequencing and microbial community diversity analysis. The sequencing platform was miseqpe250. With a minimum single sample data size of 30,000, the 16S rDNA amplification primers were 338F/806R, with a forward primer sequence of ACTCCTACGGGAGGCAGCAG and a reverse primer sequence of GGACTACHVGGGTWTCTAAT. To obtain the requisite high-quality sequences, the collected dates were screened, optimized, and concatenated, followed by quality control and the removal of questionable sequences. These high-quality sequences were clustered under 97% similarity. OTUs were obtained for species classification after chimeric screening. Finally, the abundance data in each sample’s OTUs were counted for further analysis.

### Statistics

The experimental data are manifested as mean ± standard deviation (SD) and GraphPad Prism 7.0 (GraphPad Software Inc., San Diego, CA, United States). Any differences were analyzed by one-way analysis of variance and Tukey’s multi range test and were considered significant when *p* < 0.05. Sequences were clustered using USEARCH (version 10.0) with 97% (default) similarity and we used 0.005% of the sequenced sequence count to filter OTUs. The DADA2 method in QIIME2 (version 2020.6) was used to denoise the data after quality control and 0.005% of the sequence count was used as a threshold to filter ASVS.

## Results

### Analysis of Enterotype in Volunteers

**FIGURE 1:**
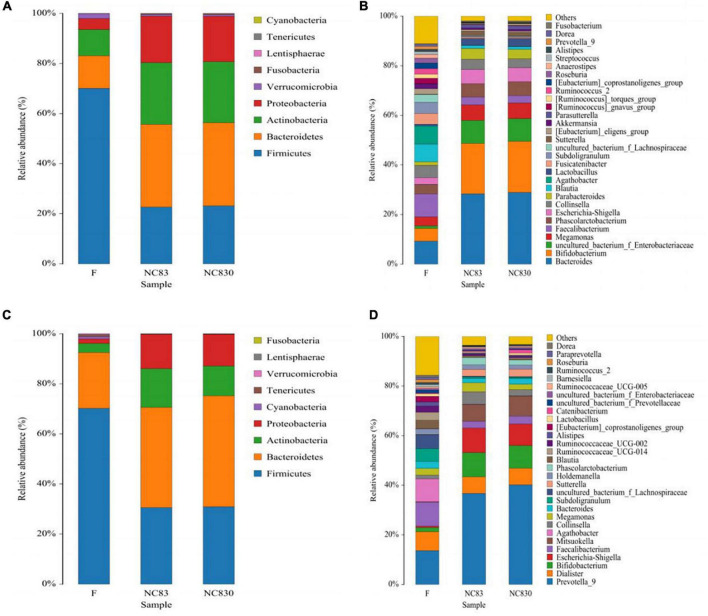
Relative abundance of gut microbiota at phylum level and genus level in different fermentation broth and different enterotype. **(A,B)** Flora composition of ET B at phylum and genus levels; **(C,D)** Flora composition of ET P at phylum and genus levels.

According to the European Molecular Biology Laboratory’s definition of enterotypes (28), there are three genus-level enterotypes. Enterotype 1 (ET B) is dominated by *Bacteroides* in the gut microbiota, enterotype 2 (ET P) is dominated by *Prevotella* in the gut microbiota, and enterotype 3 (ET F) is dominated by *Ruminococcus* in Firmicutes. ET B is engaged in the synthesis of biotin, riboflavin, pantothenic acid, and ascorbic acid, whereas ET P is involved in thiamine and folic acid synthesis. ET F is characterized by the predominance of *Ruminococcus* with mucin degradation activity and membrane transport of sugars (29). At the genus level, four human subjects’ gut microbiota was ET B (Figure 1B), whereas the other four were ET P (Figure 1D).

### Effect of PLNC8 on the Structure of Gut Microbiota

High-throughput sequencing of the number of gut bacteria in fecal samples was used to study the effect of PLNC8 on the gut microbiota. The microbial communities of three groups of fecal samples (F, NC83, and NC830) were examined using a high-throughput Illumina sequencing platform for analysis of 16S rDNA (V3 – V4 hypervariable region). Figures 1A–D shows the community composition of the phylum (A and C) and genus (B and D) levels of ET B and ET P, respectively. The dominant microbiota of ET B was *Bacteroides*, and the dominant microbiota of ET P was *Prevotella*. ET B and ET P had similar gut microbiota species at the phylum level. Both contained Firmicutes, Bacteroidetes, Actinobacteria, Proteobacteria, Fusobacteria, Verrucomicrobia, and Cyanobacteria, differing only in the abundance of their microbiota (Figures 1A,C). However, at the genus level, ET B contained *Parabacteroides*, *Fusicatenibacter*, *Akkermansia*, *Parasutterella*, *Anacrostipes*, and *Streptococcus*, genera not found in ET P. Meanwhile, ET P contained the genera *Dialister*, *Mitsuokella*, *Ruminococcaceae_*UCG*-*002, *Ruminococcaceae*_UCG-014, *Ruminococcaceae_*UCG-005, *Catenibacterium*, and *Barnesiella*, none of which were found in ET B (Figures 1B,D). In addition, at the phylum level, compared to group F, Bacteroidetes, Actinobacteria, and Proteobacteria had increased, and Verrucous and Firmicutes had decreased in the bacteriocin groups in both ET B and ET P. At the genus level, the bacteriocin groups had more *Bacteroides*, *Bifidobacterium*, *Megamonas*, *Escherichia-Shigella*, *Parabacteroides*, and *Lactobacillus* and less *Streptococcus* than group F in ET B. Furthermore, bacteriocins increased the abundance of *Prevotella*_9, *Bifidobacterium*, *Escherichia-Shigella*, *Mitsuokella*, and *Collinsella* in ET P.

### Effect of PLNC8 on Short-Chain Fatty Acids Content

**FIGURE 2:**
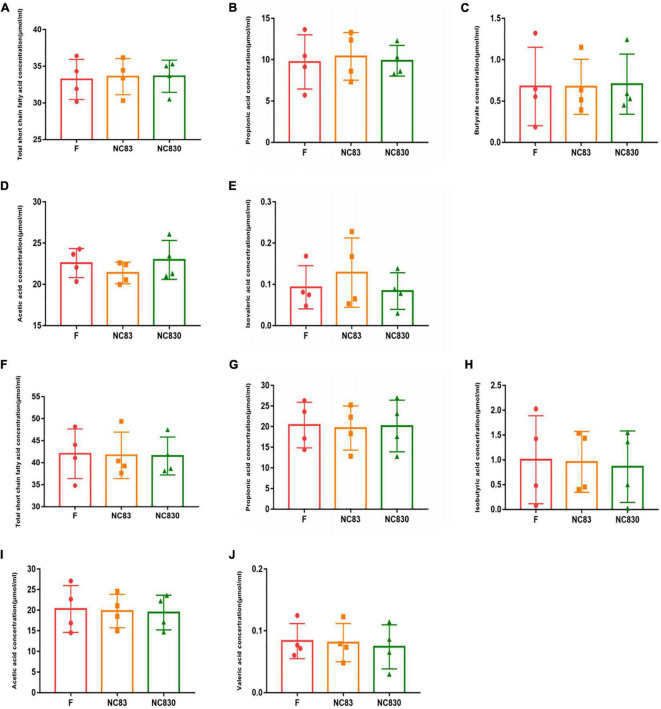
The content of SCFAs produced by the *in vitro* fermentation of PLNC8 by ET B’s volunteers **(A–E)** and ET P’s volunteers **(F–J)**. The fermentation time is 24 h, the horizontal coordinate is different media, and the vertical coordinate is concentration. An asterisk indicates *p* < 0.05. Six kinds of short chain fatty acids including acetic acid, propionic acid, butyric acid, isobutyric acid, valeric acid, and isovaleric acid were detected.

Short-chain fatty acids are major fermentation products metabolized by intestinal anerobic bacteria; they have beneficial effects on the human body (30–32). Figure 2 shows that after 24 h of fermentation, the level of SCFAs did not increase. SCFAs for ET B (Figures 2A–E) and ET P (Figures 2F–J) were not significantly different. The NC83 group exhibited a greater level of isovaleric acid than the F and NC830 groups in ET B. Furthermore, the NC830 group had a higher acetic acid level than the F and NC83 groups. However, no significant changes in SCFAs levels were seen for ET P in any of the three groups.

### Effect of PLNC8 on Bacterial Microbiota Composition

**FIGURE 3:**
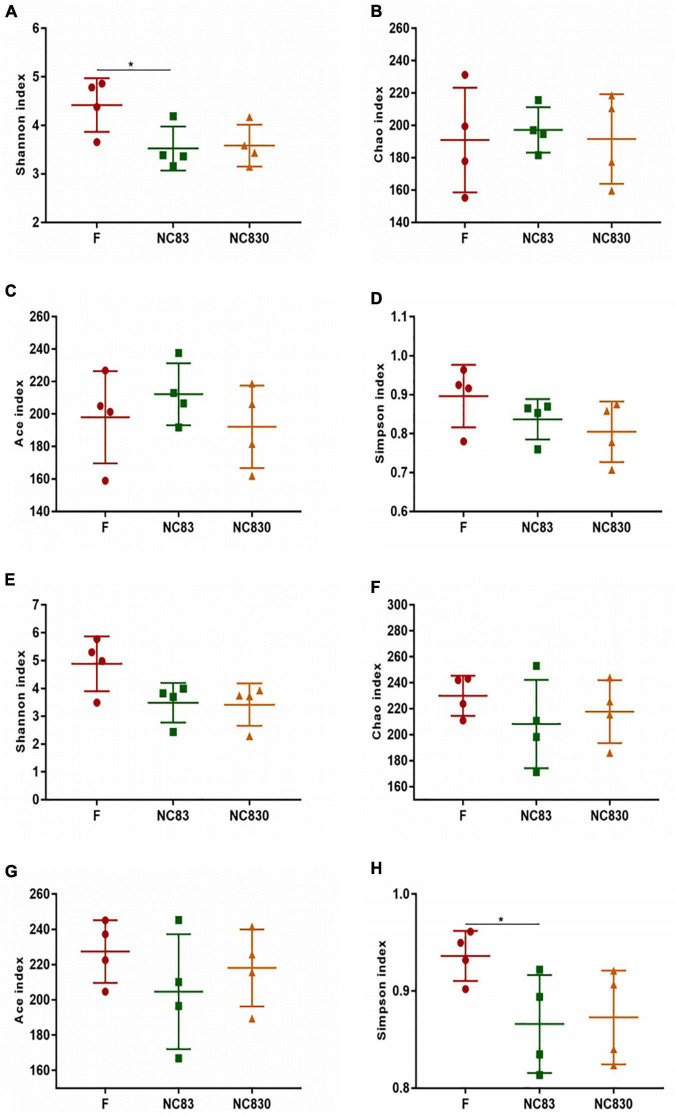
Alpha diversity index of subjects with ET B **(A–D)** and ET P **(E–H)**. Alpha diversity index includes Shannon index, Chao index, Ace index, and Simpson index. *P < 0.05.

Alpha diversity evaluates the variation of microbes in individual samples, including the Shannon, Chao, Ace, and Simpson indices (Table 1 and Figure 3; 33). Alpha diversity of ET B (Figures 3A,D) shows that the Shannon index of group F was significantly higher than that of group NC83, while the Chao, Ace, and Simpson indices were not significant between groups F, NC83, and NC830. However, ET P differed from ET B in that the Simpson index was higher in group F than in NC83 or NC830. Furthermore, the other indices did not change significantly.

**TABLE 1:** Alpha diversity of samples among different treatment groups.

Samples	Ace	Chao	Shannon	Simpson
**ETB**				
F	198.0369 ± 38.7715	199.8266 ± 31.3876	4.9022 ± 0.5223	0.9362 ± 0.03415
NC830	192.1550 ± 30.6212	192.7829 ± 25.7170	3.7865 ± 0.35315	0.8728 ± 0.04945
NC83	212.2241 ± 30.7241	199.2797 ± 16.3869	3.7281 ± 0.3657	0.8660 ± 0.52575
**ETP**				
F	234.9570 ± 12.3582	236.3214 ± 15.5714	5.3505 ± 0.3669	0.9348 ± 0.0188
NC830	227.6800 ± 11.9627	228.4508 ± 12.8794	3.7953 ± 0.1329	0.8371 ± 0.0591
NC83	207.4235 ± 40.5425	211.6323 ± 40.3823	3.8377 ± 0.1396	0.8623 ± 0.0010

*Alpha diversity means the diversity in a specific region or ecosystem. The commonly used indicators to measure flora abundance include Chao richness estimator and Ace richness estimator; Indicators for measuring flora diversity include Shannon diversity index and Simpson diversity index. Data presented as means of duplicates ± standard deviation.*

**FIGURE 4:**
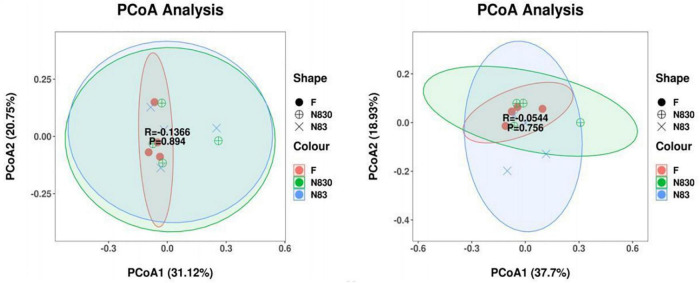
PCoA analysis based on weighted UniFrac distance. Each point represents a sample, and points of the same color came from the same treatment. The first principal com ponent is plotted on the *X*-axis, and the second principal component is plotted on the *Y*-axis. The percentage on each axis indicates the contribution to the discrepancy among samples. Beta diversity index of subjects with ET B **(A)**. Beta diversity index of subjects with ET P **(B)**.

Beta diversity analysis is used to analyze changes in species composition at temporal and spatial scales. Unweighted principal coordinate analysis (PCoA) was performed to assess the beta diversity of fermentation products in groups F, NC83, and NC830 at the genus level (34). Figures 4A,B shows the PCoA scores for ET B (A) and ET P (B). PCoA plots provided a visual representation of the similarity in bacterial community composition for all samples, with PCoA1 and PCoA2 being the principal coordinate components responsible for the differences between samples (PCoA1 and PCoA2 explanatory values were 31.12 and 20.75% for ET B and 37.7 and 18.93% for ET P, respectively). In Figure 4, the distance between the individual samples in the F group was short, indicating that the individual samples of the F group have similar bacterial compositions. Meanwhile, the distances between individual samples in the bacteriocin groups were relatively long, indicating greater variability in bacterial composition among their individual samples. However, there was some overlap between the F group and the bacteriocin groups, which suggested that under the influence of PCoA1 and PCoA2, the composition of gut microbiota in these groups was partially similar and partially different. Bacteriocins as anti-microbial peptides influence the microbial consortium (35).

**FIGURE 5:**
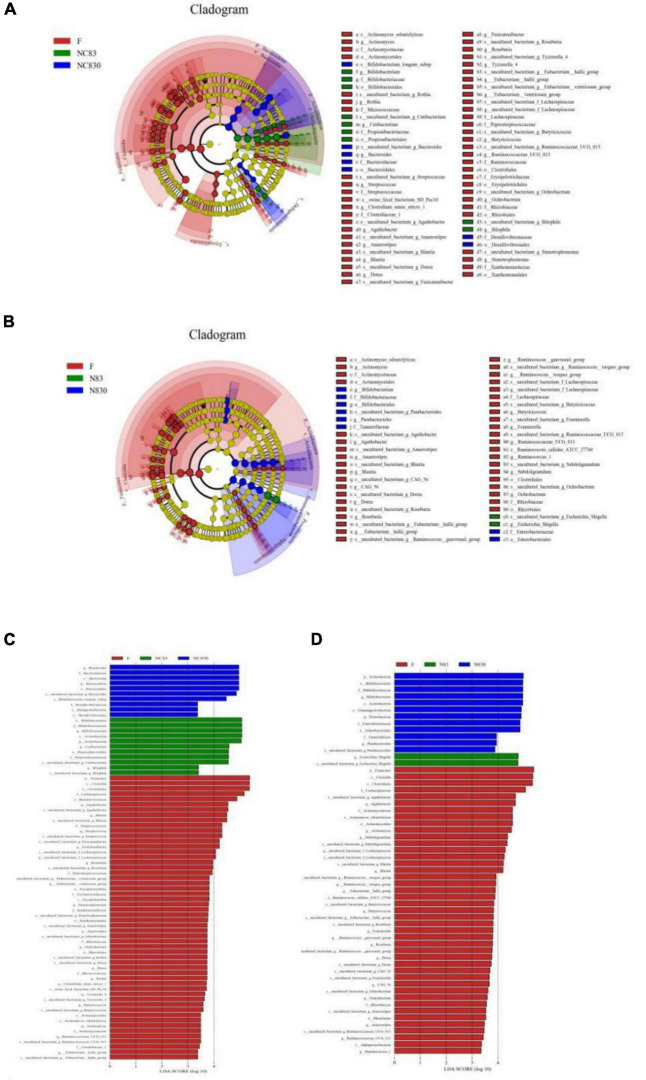
Plantaricin NC8 changed gut microbiota composition. **(A)** is ET B’s cladogram; **(B)** is ET P’s cladogram; **(C)** is ET B’s LDA effect; and **(D)** is ET P’s LDA effect. Distribution of fecal microbiota at the level of genus, phylum, family, and order. Dot size is proportional to taxon abundance. Student *t*-test was used for statistical analysis, and *p* < 0.05 is significant.

The LDA Effect Size (LEfSe) can determine the abundance of taxa between different groups. Figures 5A–D shows the LEfSe plots of the gut microbiota of ET B (A) and ET P (B), with the taxonomic classes shown to their right, in addition to the LDA plots of the gut microbiota of ET B (C) and ET P (D). LEfSe analysis (LDA threshold of 2.0) was used to compare the relative abundances of bacteria in groups. The LEfSe plots show that the microbiota was significantly higher in the two PLNC8 groups than in group F. For ET B, at the levels of phylum and class, Bacteroidetes in NC830 group and Actinobacteria in NC83 group were significantly different from those in the F group; at the order level, Bacteroidales and Desulfovibrionales were significantly enriched in the NC830 group, Bifidobacteriales and Propionibacteriales were significantly enriched in the NC83 group, Clostridiales, Erysipelotrichia, Xanthomonadales, and Coriobacteriales in the F group were significantly different from those in the other groups; at the family level, Bacteroidaceae and Desulfovibrionales in the NC830 group, and Bifidobacteriaceae and Propionibacteriales in the NC83 group were significantly enriched; at the genus level, *Bacteroides* in the NC830 group, *Bifidobacterium*, *Cutibacterium*, and *Bilophila* in the NC83 group, and *Agathobacter*, *Streptococcus*, *Stenotrophomonas*, *Anaerostipes*, *Tyzzerella*_4, and *Eubacterium_hallii*_group in the F group were significantly enriched. For ET P, at the class level, Actinobacteria and Gammaproteobacteria were significantly enriched in the NC830 group, and Clostridia and Alphaproteobacteria were significantly enriched in the F group; at the phylum level, Actinobacteria and Proteobacteria in the NC830 group were significantly different from those in the F group; at the order level, Bifidobacteriales and Enterobacteriales were enriched in the NC830 group, Clostridiales and Actinomycetales were significantly enriched in the F group; at the family level, *Bifidobacteriaceae*, *Enterobacteriaceae*, and *Tannerellaceae* in the NC830 group, *Actinomycetaceae*, *Rhizobiaceae*, and *Lachnospiraceae* in the F group were significantly enriched; at the genus level, *Bifidobacterium* and *Parabacteroides* in the NC830 group, *Escherichia Shigella* in the NC83 group, *Subdoligranulum*, *Fournierella*, and *Anaerostipes* in the F group were significantly enriched.

### Correlations Among Bacterial Communities and Short-Chain Fatty Acids

**FIGURE 6:**
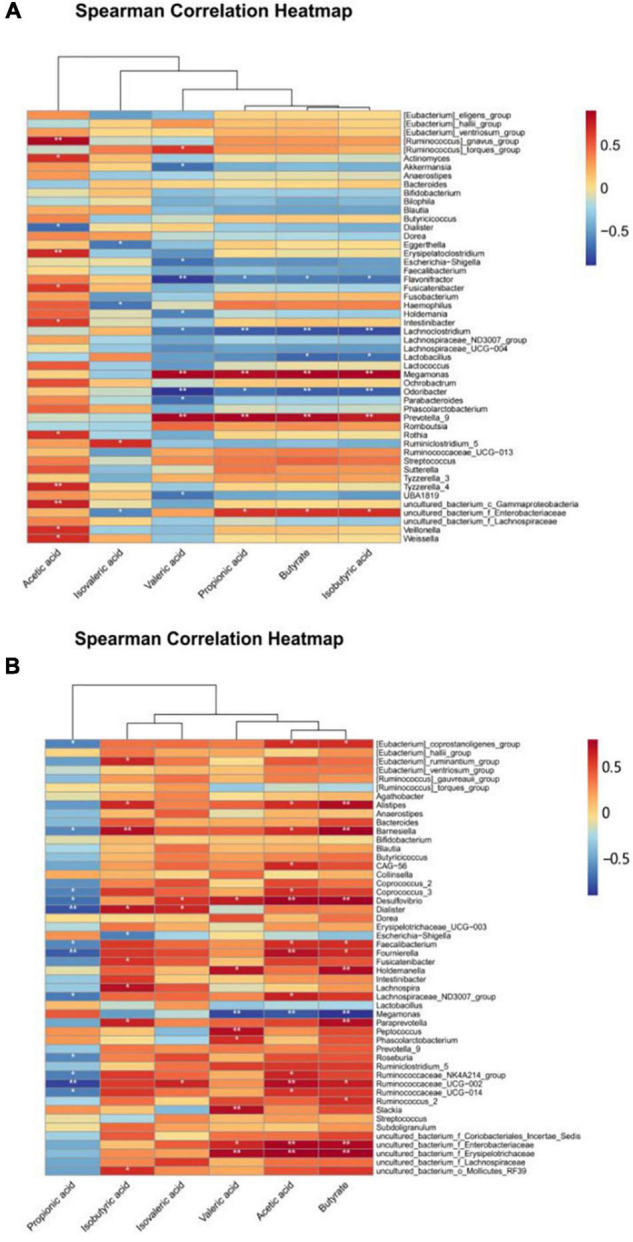
Heatmap of correlation between gut flora and SCFAs. **(A,B)** show the relationship between the intestinal flora and SCFAs of volunteers with ET B and ET P, respectively.

The correlations between strains and SCFAs were compared and assessed in accordance with the classification level. Figure 6 shows that there were significant differences in the correlation coefficients between the content of most SCFAs and species compositions. The results showed that *Ruminiclostridium_*5, *Hemophilus*, and *Eggerthella* had significantly different levels of correlation with isovaleric acid for ET B (Figure 6A). This indicates that under the exposure to different concentrations of PLNC8, these taxa in the gut microbiota can influence the production of acetic acid, valeric acid, propionic acid, butyric acid, and isobutyric acid, but they have no significant effect on isovaleric acid.

At the genus level, according to the results, *Weissella*, *Ruminiclostridium*_5, *Rothia*, *Prevotella*_9, *Odoribacter*, *Megamonas*, *Lactobacillus*, *Lachnoclostridium*, *Holdemania*, *Fusicatenibacter*, *Flavonifractor*, *Escherichia-Shigella*, *Erysipelatoclostridium*, *Eggerthella*, *Dialister*, *Akkermansia*, *Actinomyces*, *[Ruminococcus]_*torques*_*group, and *[Ruminococcus]_*gnavus_group affected the productions of SCFAs. The correlation between SCFAs and the gut microbiota of ET P was slightly different from that of ET B (Figure 6B). Propionic acid was negatively correlated with species composition, and isobutyric acid, isovaleric acid, valeric acid, acetic acid, and butyric acid were positively correlated with the gut microbiota, especially *Slackia*, *Ruminococcaceae*_UGG-002, *Ruminococcaceae*_UGG-014, *Ruminococcaceae*_2, *Prevotella*_9, *Peptococcus*, *Fournierella*, *Desulfovibrio*, *Barnesiella*, and *Alistipes*. Overall, the positive correlation between gut microbiota and SCFAs was more visible in ET P than in the other enterotypes.

## Discussion

The gut microbiota contributes to the host organism’s health. Trillions of gastrointestinal bacteria effect on local and systemic processes, such as nutrition transformation (36), vitamin supply (37), mucosal immunity maturation (38, 39), the gut brain axis (40), and even tumor progression (41). The normal functioning of the gut microbiota like that of other organs is dependent on a stable cellular composition, which in the human microbiota mostly consists of Firmicutes, Bacteroidetes, Actinobacteria, and Proteobacteria (42). Ecological dysbiosis may occur when the proportions of these groups vary drastically or when new bacterial populations emerge (43). Inflammatory bowel diseases (IBDs), autoimmune illnesses, metabolic disorders, and neurological disorders are among many health conditions linked to dysbiosis of the gut microbiota, which can lead to the formation or worsening of diseases in certain situations (44–47). The use of oral bacteriocins to treat intestinal dysbiosis is a promising field of research; however, the best dosage and route of administration for bacteriocins must be determined. The addition of nisin to the diets of mice, rabbits, chickens, and fish showed promising findings, such as a reduction in the number of pathogens or a change in the composition of the gut microbiota. *In vitro* simulations of Pediocin A, Bactofencin A, GarML, and nisin in human feces have also provided significant encouragement regarding the impact of bacteriocins on human gut microbiota. However, because of the different enterotypes among individuals, it is also important to examine how bacteriocins affect the gut microbiota of various enterotypes.

This study classified the enterotypes of eight human subjects as ET B and ET P based on the abundance of the dominant gut microbiota at the genus-pair level. There was variability in the gut microbiota structure of different enterotypes at the genus level. ET B had *Akkermansia*, *Parabacteroides*, and *Anaerostipes*, whereas ET P did not. *Akkermansia* is known to be of great value in cancer therapy and in improving host metabolic function and immune response (48). In mice fed a high-fat diet, *Parabacteroides* reduced weight gain, hyperglycemia, and hepatic steatosis (49). *Anaerostipes* produce butyric acid (50). Furthermore, *Dialister* and *Mitsuokella* were only found in ET P in this current investigation. *Dialister* was previously associated with halitosis and spondyloarthritis, but the link to gut disorders is unclear (51, 52). *Mitsuokella*, which inhibits *Salmonella* growth and invasion in pigs, could be a viable probiotic to improve food safety (53). Bacteriocins also boosted the relative abundance of beneficial bacteria, like SCFAs-producing bacteria [*Megamonas*, *Escherichia-Shigella* (54), and *Lactobacillus*], as well as probiotics [*Bifidobacterium*, *Parabacteroides* (55), *Mitsuokella*, and *Collinsella* (56)]. Overall, PLNC8 had a variable influence on the structure of the gut microbiota of ET B and ET P; it tended to increase the abundance of beneficial bacteria in different enterotypes’ samples.

The gut microbiota forms a natural protective barrier by colonizing the intestinal mucosa. This anaerobic metabolism beneficially affects daily energy demand and intestinal homeostasis (57). SCFAs are the major metabolic products of anaerobic fermentation by microbiota colonizing the mammalian gut (58), and are also linked to intestinal diseases, such as IBD (59, 60). PLNC8 did not significantly promote the synthesis of SCFAs by intestinal microorganisms in this current study, and it had little effect on the synthesis of intestinal SCFAs in ET B and ET P subjects. Although PLNC8 did not directly promote the production of SCFAs in the samples, PLNC8 enhanced the abundance of bacteria that produce SCFAs, such as *Escherichia-Shigella*, *Odoribacter*, *Lactobacillus*, *Ruminococcaceae*_UGG-002, *Ruminococcaceae*_UGG-014, *Ruminococcaceae*_2, and *Fusicatenibacter* (61, 62).

The NC83 had a more pronounced effect on the gut microbiota among the two PLNC8 fractions, according to the 16S rDNA sequencing results in this study; the bacteriostatic impact of the bacteriocin on bacteria may have played a role in this outcome (20). When *Prevotella* was the dominant bacteria, the link between SCFAs and gut microbiota was most pronounced. Furthermore, this work is the first to investigate the influence of the bacteriocin PLNC8 on gut microbiota structure and diversity using an *in vitro* fermentation model. According to our findings, the effect of various PLNC8 concentrations on the abundance of gut microbiota was not significant; the impacts on microbial species of distinct enterotypes were comparatively more evident (63). Meanwhile, various enterotypes have different albeit minor effects on the gut microbiota. Notably, PLNC8 increased the abundance of Bacteroidetes, *Bifidobacterium*, *Escherichia-Shigella*, and *Parabacteroides* and reduced the abundance of *Streptococcus* in an *in vitro* fermentation model, which is an important prerequisite for exploring how bacteriocins affect the balance and health of gut microbiota *in vivo*. This study also provides a theoretical foundation for investigating how PLNC8 affects gut microbiota balance and health *in vivo*, opening new avenues for research into the effects of additional bacteriocins on the intestine.

## Data Availability Statement

The data presented in the study are deposited in the Gene Expression Omnibus repository, accession number is GSE199735(https://www.ncbi.nlm.nih.gov/geo/query/acc.cgi?acc=GSE199735).

## Author Contributions

PL was involved in the final development of the project and manuscript preparation. JP wrote the manuscript draft, analyzed the data, and revised the article. SH was involved in part of the experiment. ML, ZC, JX, YL, HW, XZ, SL, and QG were involved in revising the language and logic of the article. All authors contributed to the article and approved the submitted version.

## Conflict of Interest

The authors declare that the research was conducted in the absence of any commercial or financial relationships that could be construed as a potential conflict of interest.

## Publisher’s Note

All claims expressed in this article are solely those of the authors and do not necessarily represent those of their affiliated organizations, or those of the publisher, the editors and the reviewers. Any product that may be evaluated in this article, or claim that may be made by its manufacturer, is not guaranteed or endorsed by the publisher.
